# Comparative interactions of withanolides and sterols with two members of sterol glycosyltransferases from *Withania somnifera*

**DOI:** 10.1186/s12859-015-0563-7

**Published:** 2015-04-16

**Authors:** Vibha Pandey, Yogeshwar Vikram Dhar, Parul Gupta, Sumit K Bag, Neelam Atri, Mehar Hasan Asif, Prabodh Kumar Trivedi, Pratibha Misra

**Affiliations:** Council of Scientific and Industrial Research, National Botanical Research Institute (CSIR-NBRI), Rana Pratap Marg, Lucknow, 226 001 India; Department of Botany, Faculty of Science, Banaras Hindu University, Varanasi, 221005 India

**Keywords:** Sterol glycosyltransferases, Withanolides, Sterols, Docking, Enzyme-substrate complex

## Abstract

**Background:**

Sterol glycosyltransferases (SGTs) are ubiquitous but one of the most diverse group of enzymes of glycosyltransferases family. Members of this family modulate physical and chemical properties of secondary plant products important for various physiological processes. The role of SGTs has been demonstrated in the biosynthesis of pharmaceutically important molecules of medicinal plants like *Withania somnifera*.

**Results:**

Analysis suggested conserved behaviour and high similarity in active sites of *Ws*SGTs with other plant GTs. Substrate specificity of *Ws*SGTs were analysed through docking performance of *Ws*SGTs with different substrates (sterols and withanolides). Best docking results of *Ws*SGTL1 in the form of stable enzyme-substrate complex having lowest binding energies were obtained with brassicasterol, transandrosteron and *Ws*SGTL4 with solasodine, stigmasterol and 24-methylene cholesterol.

**Conclusion:**

This study reveals topological characters and conserved nature of two SGTs from *W. somnifera* (*Ws*SGTs) i.e. *Ws*SGTL1 and *Ws*SGTL4. However, besides being ubiquitous in nature and with broad substrate specificity, difference between *Ws*SGTL1 and *Ws*SGTL4 is briefly described by difference in stability (binding energy) of enzyme-substrate complexes through comparative docking.

**Electronic supplementary material:**

The online version of this article (doi:10.1186/s12859-015-0563-7) contains supplementary material, which is available to authorized users.

## Background

Glycosylation is an important step in biosynthesis of various natural products that modifies the physical and chemical properties of plant metabolites. Glycosylation of metabolites have been reported to enhance their solubility and stability as well as facilitates their accumulation and storage in plant cells which ultimately determine bioactivity and bioavailability of natural product [1]. Glycosylation reaction is catalyzed by the specific enzymes, glycosyltransferases, (GTs; EC 2.4.x.y) which belong to a multigene family and responsible for glycosidic bond formation by transfer of activated glycosyl group to a nucleophilic acceptor molecule. Prior to the reaction, substrate that acts as glycosyl group donor is activated as nucleoside diphosphate sugars [2]. Acceptor molecule for the GTs are oligosaccharides, polysaccharides and glyco-conjugates of the secondary metabolites including phenolics, terpenoids, cyanohydrins, thiohydroximates, flavonoids, sterols and alkaloids [3,4].

GTs have been classified on the basis of sequence similarities into 97 families as mentioned in Carbohydrate Active Enzyme Database (http://www.cazy.org) [5]. Members of each GT family having related consensus sequence, corollary fold along with catalytic specificity [6-8]. A comprehensive survey of the GTs demonstrated to adopt either GT-A or GT-B fold. Tightly adjoining β/α/β domain of the GT-A fold form continuous central sheet with at least eight β-strands, while two Rossmann-like less tightly associated β/α/β domains “face each other” with ligand binding displays in the GT-B fold and associated with the conformational changes in relative orientation [9]. Formation of the enzyme substrate complex requires two highly conserved domain of GTs, one of which binds to the UDP-sugar and another binds to sugar acceptor. UDP sugar binding domain is also called as plant secondary product GT consensus sequence (PSPG) box [3,10,11]. Formation of the enzyme-substrate complex by GTs and UDP-sugar interaction has been described briefly in various reports [1,2,12,13].

Functional role of sterol glycosyltransferase is of great importance in medicinal plants like *Withania somnifera*, *Panax ginseng*, *Crocus sativus*, chickpea, etc. [14-17]. Comparative analysis of sterol glucosyltransferase (SGT) activity towards sterols without side chain suggests relatively higher activity in *W. somnifera* as compared to *Arabidopsis* [14]. Another study mentioned substrate specificity of purified cytosolic and membrane-bound sterol glycosyltransferase of *W. somnifera* towards different sterol substrates [18]. This medicinal plant has been reported to various pharmaceutical activities because of various glycosylated molecules synthesized in this plant [19-22] and has been a target for the genomic characterization [15,23-31].

Present study demonstrates catalytic behaviour of two characterized members of *Withania somnifera* glycosyltransferases (*Ws*SGTs), *Ws*SGTL1 and *Ws*SGTL4 among the large gene family [29]. The structural model of *Ws*SGTL1 and *Ws*SGTL4 is not available in database and is reported for the first time in this study. Analysis suggested specificity of these SGTs to specific molecules which might be important for synthesis of unique molecules with specific pharmaceutical activities. Several reports are available with detailed mechanism of UDP-glucose binding motif. But, involvement of the putative sterol binding motif in reaction mechanism has not been illustrated earlier. In the present study, along with structural model of *Ws*SGTs, we also describe the sterols/withanolides-*Ws*SGTs complexes, which require putative sterol binding domain (UDPGT) of the enzyme in order to catalyze reaction mechanism. Comparative analysis of protein followed by docking experiments performed to evaluate the comparative docking concert of proteins using AutoDock’s standard protocol.

## Methods

### Selection and sequence alignment of *Ws*SGTs

Two *Ws*SGT cDNA sequence of *W. somnifera*, *Ws*SGT*L*1 and *Ws*SGTL4 with accession number DQ356887.1 and EU342374, respectively, were selected from the work of Sharma et al. [14] and Chaturvedi et al. [15] and retrieved from NCBI nucleotide database (http://www.ncbi.nlm.nih.gov/nucleotide/). Sequence homology searches between selected *Ws*SGT proteins i.e. *Ws*SGTL1 and *Ws*SGTL4 were carried out using BLAST algorithm against protein data bank (PDB). The deduced polypeptide sequence alignment was performed using ClustalW program (http://www.ebi.ac.uk/clustalW/) while shading was done with the Boxshade 3.21 program (http://www.ch.embnet.org/software/BOX_form.html/).

### Topology alignment

Relationship of *Ws*SGTs (*Ws*SGTL1 and *Ws*SGTL4) with the known proteins in the PDB along with resemblance of functionally important binding regions of proteins were analysed through the structural similarity scores using ProBis tool (http://probis.cmm.ki.si/) [32] by inspecting their physiochemical properties. Active sites for both the proteins are determined by DogSiteScorer [http://dogsite.zbh.uni-hamburg.de/] and ProBis tool. Three dimensional structures of proteins were further used for structural topology alignment against non-redundant PDB (nr-PDB) database to check the structural similarity and conserved regions in *Ws*SGTs structure using by means of *de novo* comparisons of proteins ProBis tool. Structural superimposition was also performed using Chimera tool to find conserve structural folds [33].

### Proteins data and model preparation

Osmani et al. [13] mentioned about the few reports available with demonstration of crystal structure of any GT. Structures of *Ws*SGTL1 and *Ws*SGTL4 were modelled using GENO3D (http://geno3d-pbil.ibcp.fr) [34] server where both sequences were submitted to search template by using condition NPS@3D sequences at 95% identity in PDB database with expectation value of 1e-06 applying blosum62 matrix. From provided outputs, pdb Id 3H4T was selected as template for the structure modelling. Structural refinement of both the models was accomplished by Molecular dynamics simulation (MD) using GROMOS56 force field in GROMACS along with the SPC model for water. MDs were done using a time step of 1 fs at 300 K, under these conditions 1 ns MD was performed using GROMACS [35]. Structural modelling provided insight about mechanism of action of *Ws*SGTL1 and *Ws*SGTL4, the information of participating amino acids and clarifying the mechanism of action of interaction between *Ws*SGTL1 and *Ws*SGTL4. Maximum likelihood algorithm in MEGA4 was used to construct phylogenetic tree using neighbour joining method with 100 bootstrap values. Accession numbers of the organisms are provided in Additional file 1.

### Ligands data and preparation

The models of *Ws*SGTL1 and *Ws*SGTL4 were used to study protein-ligand conformations by automatic docking for 14 substrates including 10 sterols (β-sitosterol, brassicasterol, deactyl-16-DPA, dehydro-epiandrosteron, epoxypregnenolone, ergosterol, pregnenolone, transandrosterone, solasodine, stigmasterol and 24-methylene cholesterol) and 4 withanolides (withaferin A, withanolide A, withanolide B and withanolide D). Structure Data File (SDF) of the selected ligands were downloaded from the Pubchem (http://pubchem.ncbi.nlm.nih.gov) and further converted into PDB format using OpenBabel Tool (http://openbabel.org/wiki/Main_Page) [36].

### Docking simulations

Docking studies were performed to predict the putative modification of binding modes of group of sterols and withanolides with the structural model of *Ws*SGTL1 and *Ws*SGTL4. The grid size was set to cover both acting domains present in *Ws*SGTL1 and *Ws*SGTL4 protein with grid spacing of 0.375 Å. Genetic algorithm (GA) was applied as searching parameter with 10 number of GA runs and setting population size 150, maximum number of energy evaluations was set to 25,00,000 with considering the maximum number of generations to 27,000. Binding of *Ws*SGTs (*Ws*SGTL1 and *Ws*SGTL4) with different ligands were performed using AutoDock 4.0 (http://autodock.scripps.edu) [37]. The lowest binding energy conformation with H-Bonds in cluster was considered as the most favourable docking pose. Protein-ligand complexes obtained from AUTODOCK 4 were further viewed in UCSF-Chimera molecule viewer tool for better analysis of interaction [33]. In each case, 10 different docking arrangements were produced. The conformations obtained as result of rigid body docking were sorted by total binding energy, hydrogen bonds formed, bond lengths and close contacts between enzyme active sites.

## Results and discussion

### Evolutionary conserved nature of *WsSGT*s

**Figure 1:**
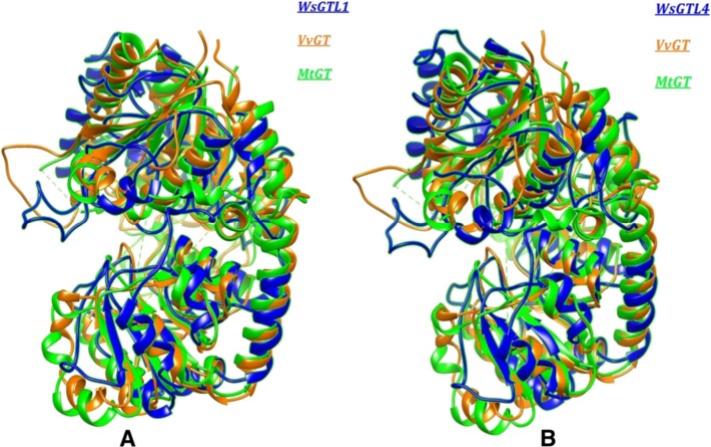
Topological alignment of *Ws*SGTs. Local structural superimposition showing similarity of *V. vinifera* GT (yellow), *M. truncatula* GT (green), with **(A)** *Ws*SGTL1 (blue) and **(B)** *Ws*SGTL4 (blue).

Structural similarities as well as conserved functional domains of *Ws*SGTs have been detected from a large database (ProBiS database) of the protein structures. Structural annotations of *Ws*SGT proteins showed some interesting features. The active site of protein is aligned with nrPDB using PROBIS tool, which aligns active site geometry with similar amino acids. The result showed that the structure of active site is very much similar to chimeric glycosyltransferase (3H4T) of *Actinoplanes teichomyceticus*, the bacterial GT with high similarity of physiochemical properties on the basis of structural equivalence for the both proteins. The structure comparison of *Ws*SGTL1 and *Ws*SGTL4 was also made for the flavonoid glycosyltransferase protein of *Vitis vinifera* (pdb 2C1X) and iso-flavonoid glycosyltransferase protein of *Medicago truncatula* (pdb 2PQ6), which demonstrates the similarity in structures as well as evolutionary conserved regions in the protein structure (Figure 1).

**Figure 2:**
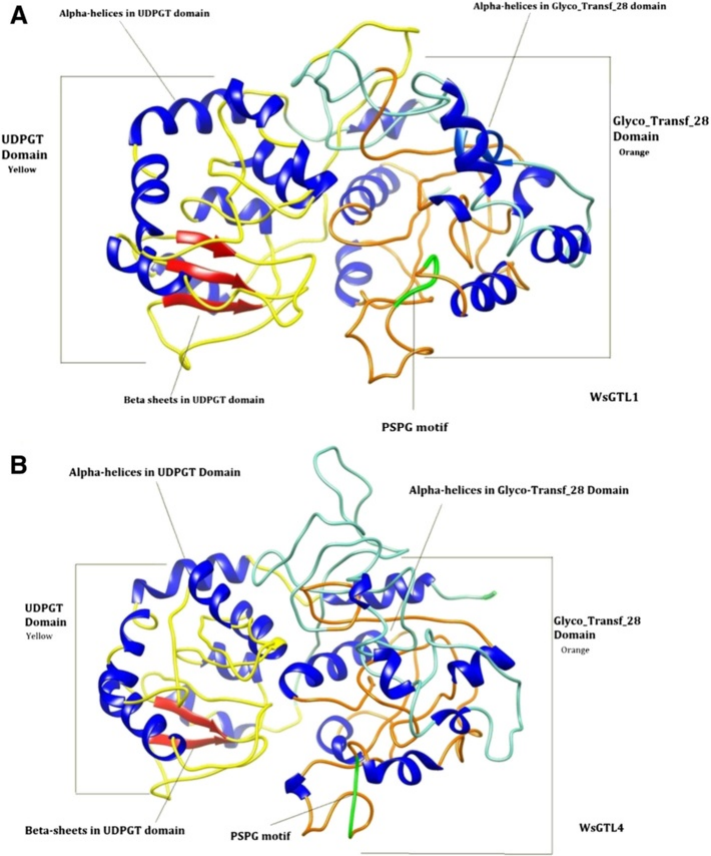
Ribbon diagrams of *Ws*SGT proteins. **(A)** *Ws*SGTL1 **(B)** *Ws*SGTL4 showing Glyco_tranf_28 domain (orange), UDPGT domain (yellow), PSPG box (green), β-sheets (red) and α-helices (blue).

On the basis of sequence similarity, more than 75% of GTs from prokaryotes to eukaryotes were grouped into three monophyletic super families named as GT-A, GT-B and GT-C. Among three, GT-A and GT-B evolved as most diverse and ubiquitous group of GTs as GT-A includes variety of organisms i.e. *E. coli*, *Bacillus subtilis*, *Bos taurus*, *Oryctolagus cuniculus*, *Mus musculus*, *Neisseria meningitides*, *Homo sapiens*, etc. [38]. Structure of *Ws*SGTL1 and *Ws*SGTL4 were modelled using homology modelling and are reported first time in this study. The proposed models show structural organization which contain 17 α-helices and 3 β-strands for the *Ws*SGTL1 (Figure 2A) and 19 α-helix and 2 β-strands for the *Ws*SGTL4 (Figure 2B). Previous studies indicated that the structure of these two domains of *Ws*SGTs is crucial for the activity and therefore the domains in both proteins were evaluated [6,12,39]. Structures of both the proteins suggested that *Ws*SGTL1 and *Ws*SGTL4 belong to the GT-B family glycosyltransferase, as in members of GT-B family all β-sheets of protein are in parallel orientation (Figure 2).

### Differences between *WsSGT*L1 and *WsSGT*L4 proteins

**Figure 3:**
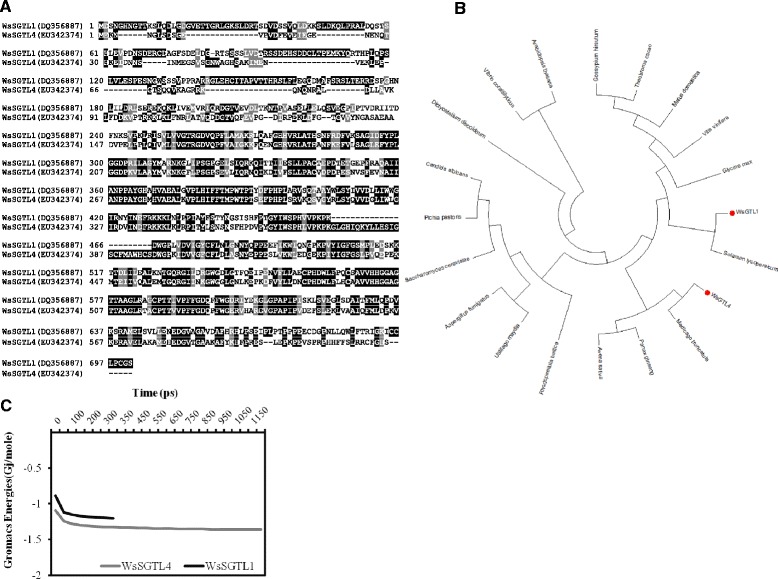
Similarity and difference between *Ws*SGTL1 and *Ws*SGTL4 proteins. **(A)** Sequence alignment of *Ws*SGTL1, *Ws*SGTL4 showing the close relationship between two enzymes. Shaded region showed highly conserved amino acids. **(B)** Gromacs energy plot of *Ws*SGTL1 and *Ws*SGTL4 proteins. **(C)** Phylogentic analysis of *Ws*SGTL1 and *Ws*SGTL4 with GTs of other organisms.

*Ws*SGTL1 (701aa) and *Ws*SGTL4 (622 aa) have variation in sequence length as well as in their structure. The sequence alignment of these two sequences shows 55% identity with 72% conservative substitution (Figure 3A). Phylogenetic analysis of both the proteins suggested that *Ws*SGTL1 was closer to *S. lycopersicum* GT, whereas *Ws*SGTL4 have more similarity to *M. truncatula* GT (Figure 3B). Phylogenetic analysis of GTs performed by Coutinho et al. and Hashimoto et al. explained the ancient origin of GTs during evolution from the time of divergence of prokaryotes and eukaryotes [9,40]. Energy minimization results for both proteins reflects that *Ws*SGTL4 seems more stable than the *Ws*SGTL1, as *Ws*SGTL1 stabilizes on −1.20 kJ/mol while *Ws*SGTL4 stabilizes on −1.58 kJ/mol (Figure 3C),

Active sites of both the protein involve UDPGT domain and a part of glyco_transf_28 domain. Comparison of active sites of these proteins indicated that cavity volume of *Ws*SGTL4 was much bigger (2371.07 Å^3^) than *Ws*SGTL1 (1481.16 Å^3^) (Table 1). Volume of cavities suggested more sensitive nature of *Ws*SGTL4 for the reaction with substrates as compared to *Ws*SGTL1. Number of H-bond donors being much more (66) in *Ws*SGTL4 as compared to *Ws*SGTL1 (38). Number of hydrophobic residues (116) is more in *Ws*SGTL4 in comparison with *Ws*SGTL1 (67 residues) as shown in Table 1.

**Table 1:** **Active site details of** ***Ws*** **SGTL1 and** ***Ws*** **SGTL4 proteins**

**Active site descriptors**	***Ws*** **SGTL1**	***Ws*** **SGTL4**
**Size and shape**		
Volume [Å^3^]	1481.16	2371.07
Surface [Å^2^]	1801.17	2989.3
Lipophilic surface [Å^2^]	1260.55	2211.65
Depth [Å]	22.86	39.6
Ellipsoid main axis ratio c/a	0.32	0.21
Ellipsoid main axis ratio b/a	0.7	0.26
Enclosure	0.05	0.07
**Functional groups**		
Hydrogen bond donors	38	66
Hydrogen bond acceptors	100	159
Metals	0	0
Hydrophobic interactions	67	116
Hydrophobicity ratio	0.33	0.34
**Elements**		
Pocket atoms	352	579
Carbon (C)	246	399
Nitrogen (N)	51	90
Oxygen	53	86
Sulphur (S)	2	4
Other elements	0	0
**Amino acid composition**		
Apolar amino acid ratio	0.55	0.52
Polar amino acid ratio	0.31	0.32
Positive amino acid ratio	0.06	0.09
Negative amino acid ratio	0.08	0.08
**Amino acids**		
ALA	10	14
ARG	1	5
ASN	4	6
ASP	2	6
CYS	2	2
GLN	2	4
GLU	4	3
GLY	7	13
HIS	3	4
ILE	1	10
LEU	9	8
LYS	1	2
MET	2	3
PHE	7	12
PRO	5	8
SER	3	4
THR	3	4
TRP	2	3
TYR	3	5
VAL	6	4
Special amino acids	0	0

### Docking of *Ws*SGTL1 and *Ws*SGTL4 with different sterols

**Figure 4:**
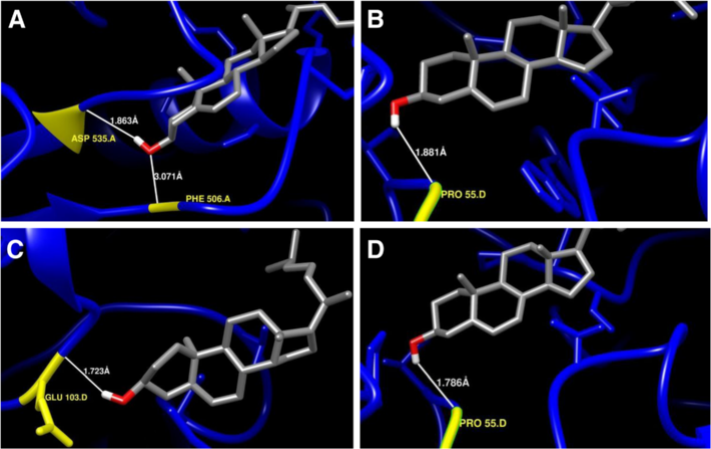
Enlarged view of interaction of *Ws*SGTL1 protein with some selected ligand molecules. **(A)** Brassicasterol, **(B)** Transandrostrone, **(C)**, 24-methylene cholesterol, and **(D)** Ergosterol. Interacting amino acid/s of protein, ‘H’ and ‘O’ atoms of hydroxyl group of interacting ligand are denoted by yellow, white and red colour, respectively.

Protein-ligand conformations by automatic docking with chosen ligands (sterols and withanolides) have been analysed using proposed model of *Ws*SGTs. For all ligands tested, the negative energies indicated a favourable interaction between the proteins and the ligands (Table 2). The obtained results revealed that the higher interaction energy was observed along with stable bonding for *Ws*SGTL1 with brassicasterol, transandrosterone, 24-methylene cholesterol, ergosterol and β-sitosterol. Stigmasterol and solasodine have similar binding energies followed by pregnenolone (Table 2). The highest affinity energy of *Ws*SGTL1 was −11.36, −9.95 and −9.75 kcal/mol for brassicasterol, transandrosterone and 24-methylene cholesterol, respectively. The best conformation of *Ws*SGTL1 was found with brassicasterol having −11.36 kcal/mol of binding energy (Figure 4A). The model revealed that ASP535 is involved in formation of H-bond with the ‘3β-OH group’ of brassicasterol and the distance between the reactive functional group of protein and ligand was 1.863 Å. Another amino acid, PHE506 of *Ws*SGTL1 formed H-Bond through –NH group reacting with oxygen of brassicasterol and the distance between the reactive functional groups of protein and ligand is 3.07 Å (Figure 4A). The second complex with the highest negative energy (−9.95 kcal/mol) of *Ws*SGTL1 is with transandrosteron, which forms a stable complex by forming H-bond with ‘O’ of PRO55 (Figure 4B). Docking pose of 24-methylene cholesterol and ergosterol is represented in Figure 4C and D, respectively.

**Table 2:** **Binding energy of selected substrates for** ***Ws*** **SGTL1 and** ***Ws*** **SGTL4 proteins**

**Ligands**	**Binding energy (kcal/mol)**	
**Sterols**	***Ws*** **SGTL1**	***Ws*** **SGTL4**
β-Sitosterol	−9.44	−8.36
Brassicasterol	−11.36	−7.97
Deactyl-16-DPA	−8.54	−8.0
Dehydroepiandrosteron	−8.28	−7.57
Epoxypregnenolone	−7.14	−6.25
Ergosterol	−9.71	−8.21
Pregnenolone	−8.97	−7.88
Solasodine	−9.42	−9.4
Stigmasterol	−9.42	−9.4
Transandrosterone	−9.95	−8.44
24-methylene cholesterol	−9.75	−8.65
**Withanolides**		
Withaferin A	−10.21	−9.14
Withanolide A	−9.28	−10.51
Withanolide B	−9.19	−9.21
Withanolide D	−9.28	−8.96

**Figure 5:**
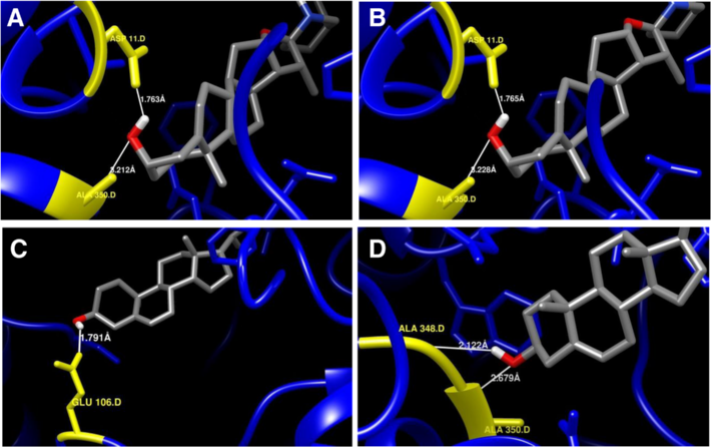
Enlarged view of interaction of *Ws*SGTL4 protein with some selected ligand molecules. **(A)** Solasodine, **(B)** Stigmastrol, **(C)** 24-methylene cholesterol, and **(D)** Transandrosterone. Interacting amino acid/s of protein, ‘H’ and ‘O’ atoms of hydroxyl group of interacting ligand are denoted by yellow, white and red colour, respectively.

For the *Ws*SGTL4 protein, the best conformations were with solasodine and stigmasterol with affinity energy of −9.40 kcal/mol for both the ligands (Table 2). The best conformations observed in solasodine and stigmasterol reveals that these two substrates follow a similar way of interaction with *Ws*SGTL proteins (Figure 5A,B). In *Ws*SGTL4, both the ligands interact in a similar manner by stabilizing the complex with 2 H-bonds with same residues ASP11 with OD2 position and ALA350 with HN position maintaining the energy value of −9.4 kcal/mol. The second most stable confirmation is with 24-methylene cholesterol followed by transandrosterone with affinity energy of 8.65and −8.44 kcal/mol (Table 2; Figure 5C,D).

In all above interactions of sterols the main functional group interacting with *Ws*SGTL proteins is ‘3β-OH group’ which indicates that it is the main active functional group in sterols. Sharma et al. and Madina et al. were also reported that the *Ws*SGTLs have affinity for the interaction with ‘3β-OH group’ of sterols [14,18]. Docking of all these sterols is restricted to UDPGT as well as glyco_transf_28 domain. Gromacs energy plots of *Ws*SGTL1 and *Ws*SGTL4 for each ligand present in Table 2 were provided in Additional files 2 and 3. Most of the sterols prefer the nonpolar hydrophobic residues ALA, PRO and VAL where ‘O’ position of these amino acids is the preferential site for binding with the 3β-OH group of sterols. 3β-OH group also show affinity for negatively charged Aspartate at ‘O’ and OD1 position for their interaction. In case of positively charged ARG, HH11 and HN positions were preferred for binding. On the other hand, clear pattern of interaction was observed by overlooking for interactions of the *Ws*SGTL4 with different sterols. Observation reveals that sterols show the tendency to bind with preferred residues with specificity for a position in some amino acids as ALA350, ALA348, ASP11, PRO200 and HIS146. *Ws*SGTL4 prefer -HN position of ALA350 residue for 3β-OH group of sterols, where in case of *Ws*SGTL1, sterols shows affinity towards the ‘O’ of ALA residue. The negatively charged Asp residue at 11th position consistently interacts with OD2 position. In all its interactions non polar cyclic residue PRO200 binds with sterols via ‘O’ position.

### Docking of *Ws*SGTL1 and *Ws*SGTL4 with withanolides

**Figure 6:**
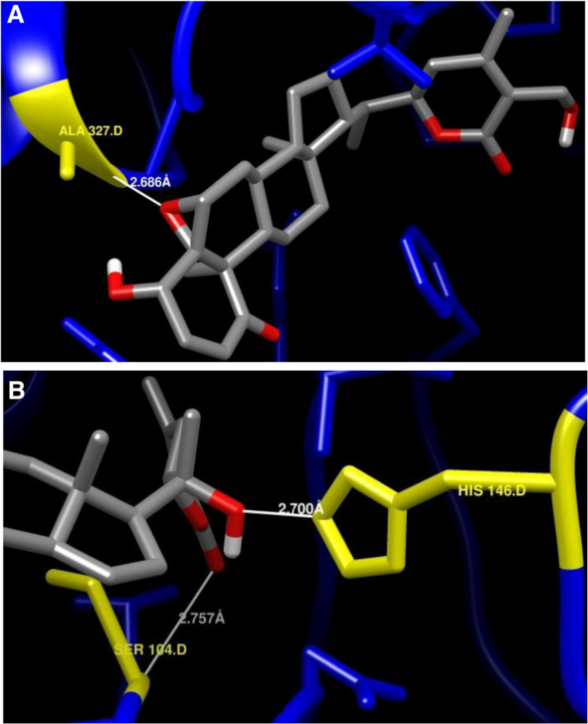
Enlarged view of interaction of *Ws*SGT proteins with some selected withanolides. **(A)** *Ws*SGTL1 with withaferin A, **(B)** *Ws*SGTL4 with withanolide A. Interacting amino acid/s of protein, ‘H’ and ‘O’ atoms of hydroxyl group of interacting ligand are denoted by yellow, white and red colour, respectively.

Withanolides might also be serving as a substrate for *Ws*SGTs, as these enzymes catalyze the biosynthesis of glycol-withanolides. It was observed that *Ws*SGTL1-withaferin-A complex generated with −10.21 kcal/mol of binding energy with the single hydrogen-bond via -HN of ALA327 with the bond length of 2.686 Å and −3.747 kcal/mol of the bond energy (Figure 6A). The distance between the interacting functional groups showed that this position is quite favourable for the stabilizing the complex through the H-bond. Binding of withanolide A reveals that it forms a significantly stable complex with *Ws*SGTL1 protein by forming 3 H-bonds with energy value of −9.28 kcal/mol involving ALA325 at ‘O’ position, ASP11 at OD1 position and ALA327 at HN position. It also reflects that it prefers hydrophobic side chain for the interaction.

Interactions of *Ws*SGTL4 with withanolides shows its affinity towards withanolide A and withanolide B with higher (negative) binding energy forming the conformations of −10.51 and −9.21 kcal/mol, respectively. Complex of withanolide A with *Ws*SGTL4 showed enzyme-substrate complex with −10.15 kcal/mol of binding energy and stabilized by 2 hydrogen-bonds (hydrogen of -NH group of SER104 and hydrogen of HIS146 residue) between withanolide A and *Ws*SGTL4 enzyme (Figure 6B). Distance between the interacting functional groups are 2.757 Å (SER104 at HN position) and 2.70 Å (HIS146 at HE2 position), which are suitable distance for the supporting H-bonds. Other higher energy conformation is of witanolide B which forms single H-bond with SER74 at OG position with binding energy of −9.21 kcal/mol. Gromacs energy plots of the *Ws*SGTL1 and *Ws*SGTL4 is provided in Additional file 4.

Withanolides, the steroidal lactones, prefer ALA327, ALA325 and ASP11 residues of *Ws*SGTL1 mainly for binding where -HN position for ALA327, ‘O’ position for ALA325 and OD1 position for ASP11 were observed for involvement in binding. In Withaferin A, the 5β- position ‘O’ molecule from steroid chain and 27th position hydroxyl group in lactone chain, participate in the reaction. In reaction of Withanolide A with *Ws*SGTL1, again the same group participates in the interaction, whereas interaction of *Ws*SGTL4 with the withanolide A involves participation of the 26th position ‘O’ as well as 22th position -OH group of the lactone chain in the complex formation. These results clearly reflect the tendency of the interaction of hydrophobic and negatively charged residues of *Ws*SGTs while in sterols, the 3β-OH group is the main interacting chemical domain. In withanolides, no such common interacting group was observed as each withanolide dock with the residues with the different chemical moieties.

## Conclusion

Glycosyltransferases are one of the largest families of enzymes which catalyze glycosylation of variety of acceptor molecules by the transfer of glycosyl moiety from activated nucleoside diphosphate sugar donar [41]. *Ws*SGTL1 and *Ws*SGTL4 differ in size as observed through sequence alignment and difference in their affinity towards different substrates. Observations collected in this study indicated that *Ws*SGTL1 and *Ws*SGTL4 interacts with different substrate and follow the different pattern of interaction. Hydrophobic amino acids as well as those with charged side chains play important role in the interaction with sterols. Results obtained in this study indicated that brassicasterol and withanolide A are the preferred substrates for *Ws*SGTL1 and *Ws*SGTL4, respectively. The interactions with different ligand molecules reveal that both proteins interact with all the mentioned ligands due to broad substrate specificity, however, have different affinity for the same substrate. The current study is predictive and needs to be confirmed experimentally using functional genomics approaches. This study shed light to understand glycosylation mechanism of sterol glycosides in plants.

## Additional files

Additional file 1:
**Accession numbers of organisms used in construction of phylogenetic tree.**
Additional file 2:
**Gromacs energy plots of** ***Ws*** **SGTL1 and** ***Ws*** **SGTL4 with sterols.**
Additional file 3:
**Gromacs energy plots of** ***Ws*** **SGTL1 and** ***Ws*** **SGTL4 with withanolides.**
Additional file 4:
**Chemical structures of sterols and withanolides.**
