# Supplementary material for: Comparative interactions of withanolides and sterols with two members of sterol glycosyltransferases from Withania somnifera
Source: BMC Bioinformatics. 2015 Apr 16;16(1):120. doi: 10.1186/s12859-015-0563-7 (PMC4407318; doi:10.1186/s12859-015-0563-7)
Supplement: Additional file 1: — Accession numbers of organisms used in construction of phylogenetic tree. [file 12859_2015_563_MOESM1_ESM.docx]

**Additional file 1:**

Accession numbers of organisms used in construction of phylogenetic tree.

AAN77910 , *Ustilago mydis*; XP_749357, *Aspergillus fumigates*; A7A179, *Saccharomyces cerevisiae*; XP_002493572*, Pichia pastoris*; AAD28546.1, *Dictyostelium discoideum*; AAD29571, *Candida albicans*; EEX31207, *Vibrio coralliilyticus*; WP_007334105, *Rhodopirellula baltica*; ABN05866, *Medicago truncatula*; BAC22616, *Panax ginseng*; AEE31974, *Arabidopsis thaliana*; CAB06081, *Avina sativa*; XP_004237799, *Solanum lycopersicum*; AHX00585, *Gossypium hirsutum*; XP_010665137, *Vitis vinifera*; XP_007019521, *Theobroma cocao*; XP_008381826, *Malus domestica*; XP_006606166, *Glycine max*
