# Supplementary figures and images for: Comparative interactions of withanolides and sterols with two members of sterol glycosyltransferases from Withania somnifera
Source: BMC Bioinformatics. 2015 Apr 16;16(1):120. doi: 10.1186/s12859-015-0563-7 (PMC4407318; doi:10.1186/s12859-015-0563-7)

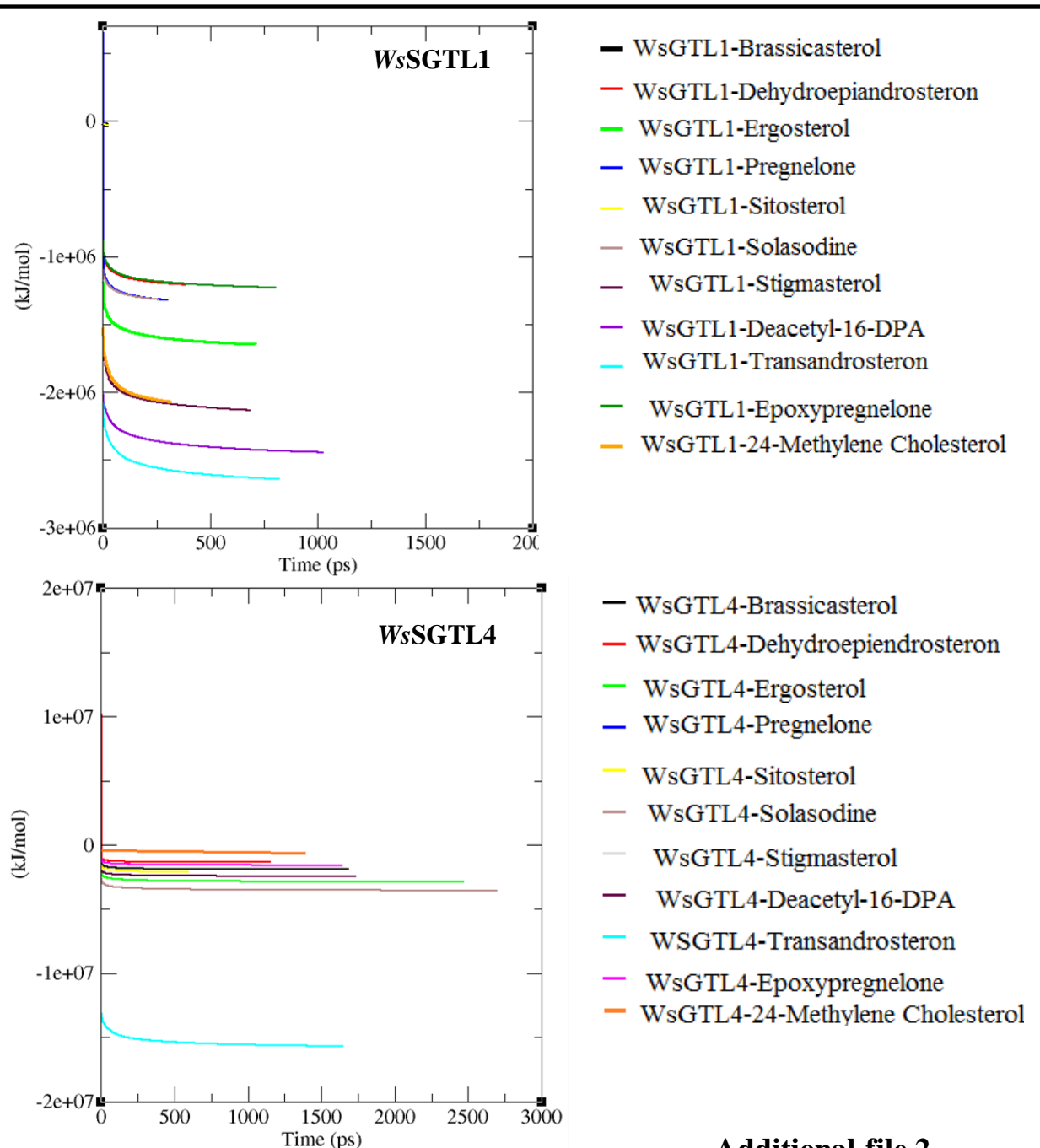

Supplement: Additional file 2: — Gromacs energy plots of Ws SGTL1 and Ws SGTL4 with sterols. [file 12859_2015_563_MOESM2_ESM.pdf]

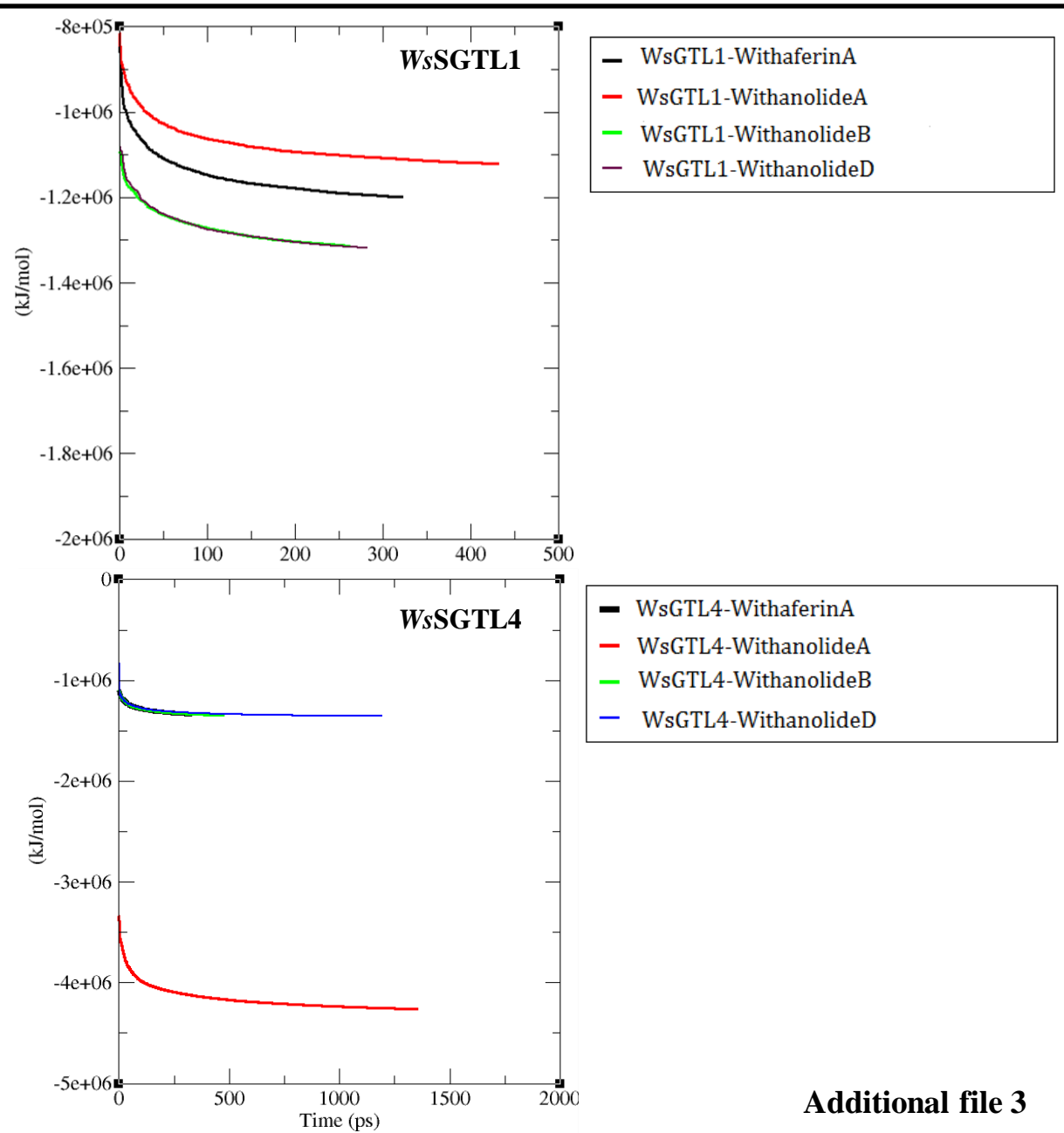

Supplement: Additional file 3: — Gromacs energy plots of Ws SGTL1 and Ws SGTL4 with withanolides. [file 12859_2015_563_MOESM3_ESM.pdf]
