# Supplementary material for: Comparative interactions of withanolides and sterols with two members of sterol glycosyltransferases from Withania somnifera
Source: BMC Bioinformatics. 2015 Apr 16;16(1):120. doi: 10.1186/s12859-015-0563-7 (PMC4407318; doi:10.1186/s12859-015-0563-7)
Supplement: Additional file 4: — Chemical structures of sterols and withanolides. [file 12859_2015_563_MOESM4_ESM.pdf]

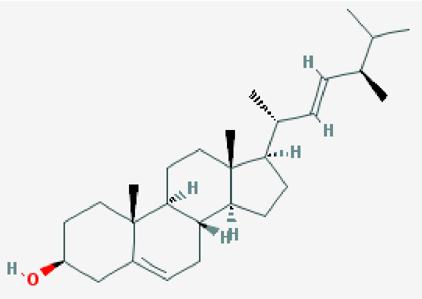

Brassicasterol

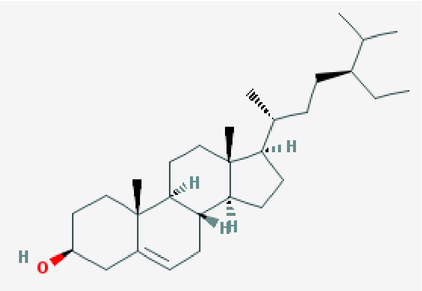

B-sitosterol

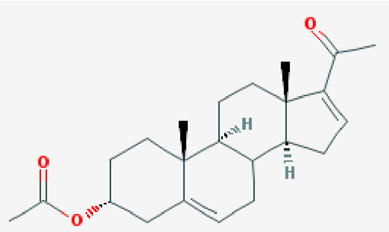

16-DPA

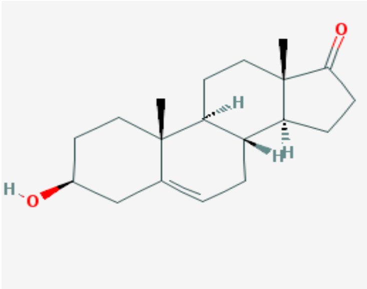

Dehydro epiandrosteron

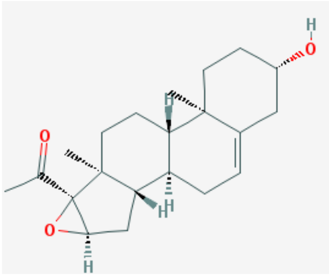

Epoxypregnenolene

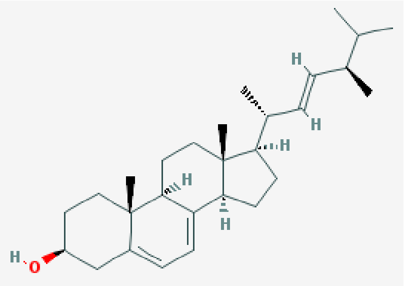

Ergosterol

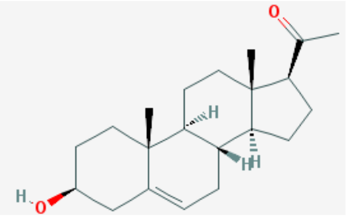

Pregnenolone

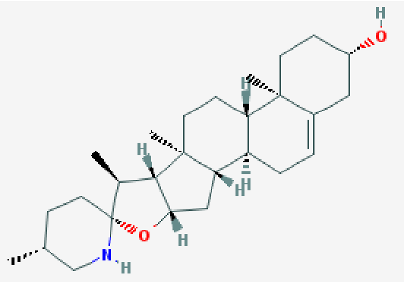

Solasodine

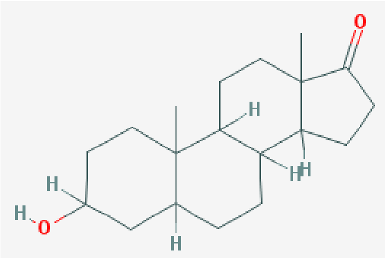

Transandrosterone

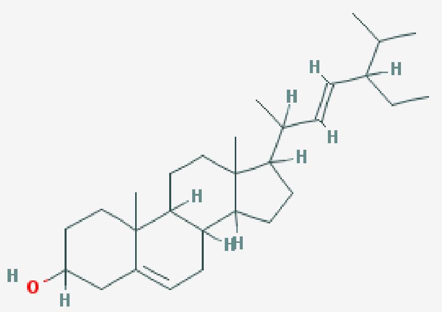

Stigmasterol

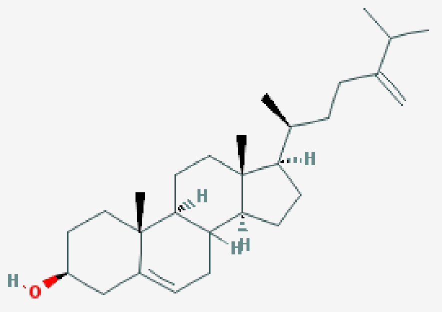

24-methylene cholesterol

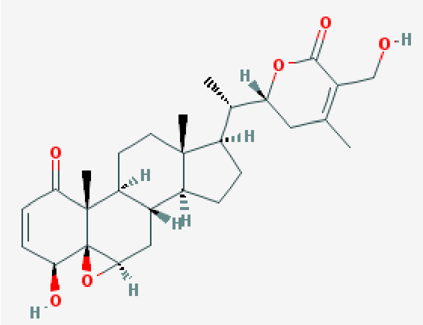

Withaferin A

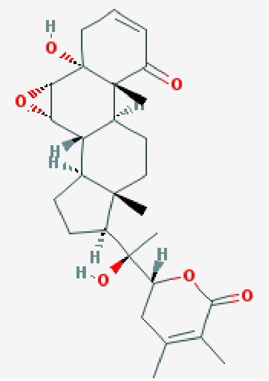

Withanolide A
